# The association of *miR34b/c* and *TP53* gene polymorphisms with Wilms tumor risk in Chinese children

**DOI:** 10.1042/BSR20194202

**Published:** 2020-02-28

**Authors:** Juxiang Wang, Susu Lou, Xiaokai Huang, Yixiao Mo, Zhen Wang, Jinhong Zhu, Xiaoqian Tian, Jiandong Shi, Haixia Zhou, Jing He, Jichen Ruan

**Affiliations:** 1 Department of Hematology, The Second Affiliated Hospital and Yuying Children’s Hospital of Wenzhou Medical University, Wenzhou 325027, Zhejiang, China; 2 Department of Clinical Laboratory, Biobank, Harbin Medical University Cancer Hospital, Harbin 150040, Heilongjiang, China; 3 Department of Pediatric Surgery, Guangzhou Institute of Pediatrics, Guangdong Provincial Key Laboratory of Research in Structural Birth Defect Disease, Guangzhou Women and Children’s Medical Center, Guangzhou Medical University, Guangzhou 510623, Guangdong, China

**Keywords:** miR-34b/c, polymorphism, susceptibility, TP53, Wilms tumor

## Abstract

Wilms tumor is the most common pediatric malignancy in the kidney. The *miR34b/c* is a downstream target gene of the transcription factor p53. The important role of *TP53* mutations, the methylation of *miR34b/c*, and the interaction between these two molecules in tumorigenesis have been well documented. Due to the biological connection between p53 and *miR34b/c*, in the present study, we investigated the association between polymorphisms in these two molecules and Wilms tumor susceptibility through genotyping two important functional polymorphisms (*miR34b/c* rs4938723 T>C and *TP53* rs1042522 C>G) in 183 cases and 603 controls. Adjusted odds ratios (ORs) and 95% confidence intervals (CIs) derived from the logistic regression analysis were used to assess the correlation of *miR34b/c* rs4938723 and *TP53* rs1042522 polymorphisms with Wilms tumor risk. Our results indicated that the association of *miR34b/c* rs4938723 and *TP53* rs1042522 polymorphisms with Wilms tumor susceptibility was not statistically significant. Stratified analysis by age, gender, and clinical stage, as well as combined effect analysis were also performed, yet, no significant association was found. In conclusion, our study indicated a lack of association between the two selected polymorphisms and Wilms tumor susceptibility. Our findings need to be verified in studies with larger sample size in the future.

## Introduction

Wilms tumor, a malignant kidney tumor in children, currently has achieved a high survival rate in different geographical regions [1,2]. It is reported that approximately 98% of Wilms tumor are sporadic [3]; and approximately 80 percent of Wilms tumor patients are diagnosed before 5 years old [4]. Molecular genetics studies showed that Wilms tumor had a complex etiology involving genetic lesions in the multiple sites [5,6]; and various gene polymorphisms play pivotal roles in the occurrence of Wilms tumor. However, there remain numerous functional single nucleotide polymorphisms (SNPs) in oncogenes and tumor suppressor genes, whose roles in Wilms tumor susceptibility need to be clarified.

As we know, gene mutation is a crucial factor in the tumorigenesis of Wilms tumor. For example, mutations in the coding sequence of the *p53* gene were found in Wilms tumor patients by several research groups [7,8]. Apart from that, Slade et al. [9] showed that translocation t(5;6)(q21;q21), leading to the inactivation of the *HACE1* gene, predisposed Wilms tumor. Moreover, over the past decade, many genes have been found to be involved in the development of Wilms tumor, including *MYCN* [10], *CTNNB1* [11], *WTX* [12], *CHEK2* [6], *BARD1* [13], *hOGG1* and *FEN1* gene [14] and *KRAS* [15].

MicroRNAs are recognized as endogenous non-coding RNAs that can play different biological roles by targeting related genes [16]. In addition, microRNAs are considered promising biomarkers for their stability and ease of detection [17]. *miR34b/c*, a recognized tumor-suppressor gene, was found to be methylated in a variety of tumors [18]. Among the mir-34 family, *miR34b/c* has a stronger ability to inhibit tumor growth, which not only enhanced the attachment of tumor cells, but also inhibited the growth and invasion of cells [19]. On one hand, *miR34b/c* is a mediator of the P53 signaling network and on the other hand, *TP53* can regulate the transcription of *miR34b/c* [20]. The rs4938723 T>C polymorphism, situated in the *miR34b/c* promoter region, influenced the expression of *miR34b/c* by affecting the binding efficiency of GATA-X transcription factor [21].

*TP53* is a tumor suppressor, which inhibits tumorigenesis mainly by regulating gene transcription [22]. Mutations in *TP53* are common in the most cancer types and have been the focus of various research groups. The *TP53* can facilitate DNA repair by stalling the cell cycle in the G_1_ phase when DNA damage occurs [23]. Moreover, Franken et al. [24] reported that the expression of p53 protein was positively correlated with tumor invasion. *TP53* rs1042522 C>G polymorphism, the polymorphic variant in *TP53* codon 72, has been shown to be associated with susceptibility to a variety of tumors. *miR34b/c* cooperates with *TP53* to control tumorigenesis. In the *TP53*-deficient cells, *miR34b/c* can overexpress to compensate the functions of p53, such as the regulation of senescence and apoptosis [25]. However, the combined effects of *miR34b/c* rs4938723 T>C and *TP53* rs1042522 C>G on Wilms tumor susceptibility remained unclarified. Thus, we conducted the present study to explore the association of *miR34b/c* rs4938723 T>C and *TP53* rs1042522 C>G polymorphisms with Wilms tumor risk.

## Materials and methods

### Study subjects

In the present study, we recruited 183 Wilms tumor patients and 603 cancer-free volunteers from Yuying Children’s Hospital of Wenzhou Medical University and Guangzhou Women and Children’s Medical Center [26,27]. The age, gender, and clinical stage of all participants are detailed in Supplementary Table S1. At the time of recruitment, all participants or their patients signed informed consent forms. In addition, this investigation was performed with the permission of the Institutional Review Boards of the Yuying Children’s Hospital of Wenzhou Medical University and Guangzhou Women and Children’s Medical Center.

### SNP selection and genotyping

In this research, we selected two most frequently investigated SNPs in *miR34b/c* and *TP53* (*miR34b/c* rs4938723 and *TP53* rs1042522 polymorphisms) to investigate their associations with Wilms tumor susceptibility. TaqMan method was used for genotyping [28–30]. In addition, negative controls were included in genotyping. In order to ensure the accuracy of genotyping, we blindly took 10% samples for repeated testing, and the results were 100% consistent.

### Statistical analysis

The Hardy–Weinberg equilibrium (HWE) of control subjects was estimated by a goodness-of fit χ^2^ test. The logistic regression analysis was performed. Adjusted odds ratios (ORs) as well as 95% confidence intervals (CIs) were used to assess the correlation of *miR34b/c* rs4938723 and *TP53* rs1042522 polymorphisms with Wilms tumor risk. We considered the difference to be statistically significant only when *P*<0.05.

## Results

### Correlation of *miR34b/c* rs4938723 and *TP53* rs1042522 polymorphisms with Wilms tumor susceptibility

The detailed results are summarized in the Table 1. Both the polymorphisms were in agreement with the HWE (*P*=0.459 for the *miR34b/c* rs4938723 T>C polymorphism, *P*=0.533 for the *TP53* rs1042522 C>G polymorphism). In our result, for *miR34b/c* rs4938723, the carriers with TC (adjusted OR = 0.73, 95% CI = 0.51–1.06, *P*=0.097) or CC (adjusted OR = 1.42, 95% CI = 0.83–2.43, *P*=0.202) genotypes showed no significant association with Wilms tumor risk compared with carriers of the TT genotype. Besides, no significant association was found between rs4938723 and Wilms tumor risk under the additive (adjusted OR = 1.02, 95% CI = 0.79–1.32, *P*=0.880), dominant (adjusted OR = 0.85, 95% CI = 0.60–1.19, *P*=0.345) or recessive models (adjusted OR = 1.63, 95% CI = 0.98–2.73, *P*=0.061). For *TP53* rs1042522, the carriers with CG (adjusted OR = 1.51, 95% CI = 1.00–2.29, *P*=0.051) or GG (adjusted OR = 1.11, 95% CI = 0.66–1.85, *P*=0.700) genotypes showed no significant association with Wilms tumor risk compared with carriers of the CC genotype. Besides, no significant association was found between rs1042522 and Wilms tumor risk under the additive (adjusted OR = 1.07, 95% CI = 0.84–1.37, *P*=0.584), dominant (adjusted OR = 1.39, 95% CI = 0.93–2.06, *P*=0.109) or recessive models (adjusted OR = 0.84, 95% CI = 0.55–1.28, *P*=0.416). In summary, neither of the two selected polymorphisms showed significant association with Wilms tumor risk.

**Table 1:** Association between *miR34b/c* rs4938723 T>C and *TP53* rs1042522 C>G polymorphisms with Wilms tumor susceptibility

Genotype	Cases (*n*=170)	Controls (*n*=600)	*P*^1^	Crude OR (95% CI)	*P*	Adjusted OR (95% CI)^2^	*P*^2^
miR34b/c rs4938723 T>C (HWE = 0.459)							
TT	86 (50.59)	279 (46.50)		1.00		1.00	
TC	60 (35.29)	266 (44.33)		0.73 (0.51–1.06)	0.098	0.73 (0.51–1.06)	0.097
CC	24 (14.12)	55 (9.17)		1.42 (0.83–2.42)	0.205	1.42 (0.83–2.43)	0.202
Additive			0.881	1.02 (0.79–1.32)	0.881	1.02 (0.79–1.32)	0.880
Dominant	84 (44.91)	321 (53.50)	0.346	0.85 (0.60–1.19)	0.346	0.85 (0.60–1.19)	0.345
Recessive	146 (85.88)	545 (90.83)	0.060	1.63 (0.98–2.72)	0.062	1.63 (0.98–2.73)	0.061
TP53 rs1042522 C>G (HWE = 0.533)							
CC	39 (22.94)	175 (29.17)		1.00		1.00	
CG	98 (57.65)	291 (48.50)		1.51 (1.00–2.29)	0.052	1.51 (1.00–2.29)	0.051
GG	33 (19.41)	134 (22.33)		1.11 (0.66–1.85)	0.704	1.11 (0.66–1.85)	0.700
Additive			0.102	1.07 (0.84–1.36)	0.587	1.07 (0.84–1.37)	0.584
Dominant	131 (77.06)	425 (70.83)	0.110	1.38 (0.93–2.06)	0.111	1.39 (0.93–2.06)	0.109
Recessive	137 (80.59)	466 (77.67)	0.415	0.84 (0.55–1.28)	0.415	0.84 (0.55–1.28)	0.416

1 χ^2^ test for genotype distributions between Wilms tumor patients and controls.

2 Adjusted for age and gender.

### Stratification analysis

We then analyzed the correlation of *miR34b/c* rs4938723 and *TP53* rs1042522 polymorphisms with Wilms tumor susceptibility in subgroups stratified by age, gender and clinical stage (Table 2). However, no significant association was observed.

**Table 2:** Stratification analysis of risk genotypes with tumor susceptibility

Variables	rs4938723 (cases/controls)		OR (95% CI)	*P*	AOR (95% CI)^1^	*P*^1^	rs1042522 (cases/controls)		OR (95% CI)	*P*	AOR (95% CI)^1^	*P*^1^
	TT/TC	CC					CC	CG/GG				
Age, months												
≤18	64/247	9/21	1.65 (0.72–3.79)	0.234	1.64 (0.71–3.75)	0.244	11/80	59/188	1.79 (0.95–3.40)	0.073	1.77 (0.94–3.36)	0.079
>18	82/298	15/34	1.60 (0.83–3.09)	0.157	1.63 (0.85–3.15)	0.143	25/95	72/237	1.15 (0.69–1.93)	0.584	1.16 (0.69–1.93)	0.579
Gender												
Female	67/244	9/21	1.56 (0.68–3.57)	0.291	1.56 (0.68–3.57)	0.291	21/79	55/186	1.11 (0.63–1.96)	0.713	1.11 (0.63–1.96)	0.712
Male	79/301	15/34	1.68 (0.87–3.24)	0.121	1.68 (0.87–3.24)	0.121	18/96	76/239	1.70 (0.96–2.99)	0.067	1.70 (0.96–2.99)	0.067
Clinical stages												
I+II	58/545	7/55	1.20 (0.52–2.75)	0.673	1.25 (0.54–2.88)	0.606	15/175	50/425	1.37 (0.75–2.51)	0.304	1.38 (0.75–2.52)	0.300
III+IV	75/545	13/55	1.72 (0.90–3.29)	0.103	1.73 (0.90–3.31)	0.102	22/175	66/425	1.24 (0.74–2.07)	0.420	1.23 (0.74–2.06)	0.431

1 Adjusted for age and gender, without the corresponding stratifying factor.

AOR: adjusted odds ratio

### Combined effect analysis

As shown in the Table 3, no statistically significant association was detected in the combined effect analysis. However, the individuals with both *miR34b/c* rs4938723 TC genotype and *TP53* rs1042522 CC genotype might have a borderline significantly decreased risk of Wilms tumor (adjusted OR = 0.51, 95% CI = 0.25–1.01, *P*=0.053).

**Table 3:** The association of combined genotypes of *miR34b/c* rs4938723 T>C and *TP53* rs1042522 C>G polymorphisms with Wilms tumor risk

Genotypes		Cases (*n*=170)	Controls (*n*=600)	OR (95% CI)	*P*	AOR (95% CI)^1^	*P*^1^
rs4938723	rs1042522						
TT	CC	22 (12.94)	89 (14.83)	1.00		1.00	
TT	CG	46 (27.06)	128 (21.33)	0.95 (0.57–1.58)	0.828	0.95 (0.57–1.59)	0.837
TT	GG	18 (10.59)	62 (10.33)	0.76 (0.40–1.47)	0.417	0.77 (0.40–1.47)	0.425
TC	CC	14 (8.24)	73 (12.17)	0.50 (0.25–1.01)	0.052	0.51 (0.25–1.01)	0.053
TC	CG	35 (20.59)	137 (22.83)	0.67 (0.39–1.15)	0.147	0.67 (0.39–1.15)	0.148
TC	GG	11 (6.47)	56 (9.33)	0.52 (0.24–1.10)	0.086	0.52 (0.24–1.10)	0.087
CC	CC	3 (1.76)	13 (2.17)	0.61 (0.16–2.26)	0.456	0.61 (0.16–2.27)	0.461
CC	CG	17 (10.00)	26 (4.33)	1.72 (0.83–3.55)	0.143	1.73 (0.84–3.57)	0.140
CC	GG	4 (2.35)	16 (2.67)	0.66 (0.21–2.10)	0.479	0.66 (0.21–2.10)	0.476

1 Obtained in logistic regression models with adjustment for age and gender.

## Discussion

We conducted this case–control study to explore the correlation of *miR34b/c* rs4938723 and *TP53* rs1042522 polymorphisms with Wilms tumor risk. No significant association was found in the single locus and stratified analyses. However, the combine effect analysis suggests that the combination of these two gene polymorphisms may have a potential influence on Wilms tumor susceptibility.

To our knowledge, the association between *miR34b/c* and Wilms tumor susceptibility has not been investigated. *miR34b/c* has been shown to be intimately connected to the development and prognosis of a variety of tumors, including gastric cancer, bladder cancer, pancreatic cancer, ovarian cancer, prostate cancer, hepatocellular carcinoma, neuroblastoma and many others [31–35]. In our previous study, we found that *miR34b/c* rs4938723 T>C polymorphism has a protective effect on neuroblastoma [36]. Sato et al. [37] reported that *miR34b/c* methylation in circulating DNA could be used to predict disease progression in malignant pleural mesothelioma patients. Moreover, in a meta-analysis, *miR34b/c* gene polymorphism was shown to increase the risk of gastric cancer and liver cancer while decreasing the risk of esophageal squamous cell carcinoma and colorectal cancer [38].

*TP53* rs1402522 is one of the most investigated *TP53* gene polymorphisms. However, we found no significant association between the *TP53* rs1402522 polymorphism and Wilms tumor risk. A study by Liu et al. [39] revealed that the effect of *TP53* rs1402522 polymorphism on Wilms tumor risk is very weak, which is consistent with our result. Another study conducted by Fu et al. [40] also showed no significant association between *TP53* rs1402522 polymorphism and Wilms tumor risk, and they found that CG/GG genotypes carriers significantly increased Wilms tumor risk in children younger than 18 months of age, compared with CC genotype carriers. Moreover, it is reported that *TP53* rs1042522 may not confer susceptibility to cancers, such as neuroblastoma [41], retinoblastoma [42]. *miR34b/c* is involved in the *TP53* pathway, and their ‘double hit’ (methylation of *miR34* and *TP53* mutations) has been shown to affect survival in a variety of tumors [43]. The negative result in the current study might result from the small sample size.

Several limitations should be addressed. First, in the process of tumorigenesis, the interaction among various gene polymorphisms is very complex. Only selecting two gene polymorphisms might lead to false-negative results. Second, we did not recruit enough participants for the present study. The sample size might not be adequate to detect real association if it is weak. Third, all the recruiters were from Guangzhou and Wenzhou. As a result, selection bias might exist. Our results should be explained with caution.

In summary, our statistical results showed that the association of *miR34b/c* rs4938723 and *TP53* rs1042522 polymorphisms with Wilms tumor susceptibility was not statistically significant. Our findings require validation in the studies with larger sample size.

## Supplementary Material

Supplementary Table S1

## Abbreviations

CI: confidence interval
HWE: Hardy–Weinberg equilibrium
OR: odds ratio
SNP: single nucleotide polymorphism
